# Supplementary material for: Pulmonary mesenchymal stem cells are engaged in distinct steps of host response to respiratory syncytial virus infection
Source: PLoS Pathog. 2021 Jul 28;17(7):e1009789. doi: 10.1371/journal.ppat.1009789 (PMC8351988; doi:10.1371/journal.ppat.1009789)
Supplement: S1 Table — (DOCX) [file ppat.1009789.s008.docx]

| **RSV *versus* Ctrl 6 days p.i.** | | | | | | | |
| --- | --- | --- | --- | --- | --- | --- | --- |
| **Gene ID** | **Gene Symbol** | **Log2 Fold Change** | **P value** | **P_adj_** | **Alternative name & function** | **Biological pathway** | **Ref.** |
| ENSOARG00000020741 | TNFSF10 | 1.46 | 1.43E-09 | 1.10E-05 | TRAIL  pro-apoptotic | Apoptosis | [1, 2] |
| ENSOARG00000010042 | STC1 | 1.21 | 3.66E-06 | 1.00E-02 | Stanniocalcin-1  pro-apoptotic | Apoptosis | [3] |
| ENSOARG00000014648 | RSAD2 | 1.55 | 3.89E-11 | 7.44E-07 | Viperin  ISG | IFN pathway | [4] |
| ENSOARG00000007233 | ISG15 | 1.61 | 1.73E-09 | 1.10E-05 | ISG | IFN pathway | [5] |
| ENSOARG00000025182 | BST-2A | 1.40 | 3.38E-09 | 1.62E-05 | ISG  ISG | IFN pathway | [6] |
| ENSOARG00000013440 | IFI44 | 1.37 | 4.11E-08 | 1.57E-04 | ISG | IFN pathway | [7] |
| ENSOARG00000002881 | OASL* | 1.33 | 5.81E-07 | 1.85E-03 | ISG | IFN pathway | [8] |
| ENSOARG00000014800 | IFIT3 | 1.29 | 4.68E-06 | 1.12E-02 | RIG-G  ISG | IFN pathway | [9] |
| ENSOARG00000015177 | IFIT1 | 1.26 | 6.92E-06 | 1.32E-02 | ISG | IFN pathway | [10] |
| ENSOARG00000016787 | BST-2B | 1.03 | 6.53E-06 | 1.32E-02 | ISG | IFN pathway | [6] |
| ENSOARG00000001138 | HERC6* | 1.25 | 1.35E-05 | 2.15E-02 | Linked to ISG15 activation | IFN pathway | [11] |
| ENSOARG00000001815 | IRF* | 1.24 | 1.30E-05 | 2.15E-02 | IFN pathway | IFN pathway | [12] |
| ENSOARG00000013421 | IFI44L | 1.12 | 1.63E-05 | 2.40E-02 | ISG | IFN pathway | [7, 13] |
| ENSOARG00000014413 | IFI27L2* | 1.10 | 3.18E-05 | 4.35E-02 | ISG12b  ISG | IFN pathway | [14] |
| ISG, interferon (IFN)-stimulated gene. | | | | | | | |
| **RSV *versus* Ctrl 42 days p.i.** | | | | | | | |
| **Gene ID** | **Gene Symbol** | **Log2 Fold Change** | **P value** | **P_adj_** | **Alternative name & function** | **Biological pathway** | **Ref.** |
| ENSOARG00000009964 | TRPC4 | 1.15 | 4.84E-05 | 2.74E-02 | Calcium channel Endothelial permeability Vasodilation | Angiogenesis  Endothelium  Calcium | [15] |
| ENSOARG00000001060 | KDR | 1.10 | 1.54E-04 | 4.82E-02 | VEGF Receptor 2 (VEGFR2) | Angiogenesis  Endothelium  Calcium | [16] |
| ENSOARG00000003922 | MXRA8 | 0.99 | 7.96E-06 | 9.27E-03 | DICAM  Angiogenesis  Cartilage formation | Angiogenesis  Endothelium  Calcium | [17] |
| ENSOARG00000000498 | ITGA9 | 0.93 | 1.21E-04 | 4.49E-02 | Integrin α9  Receptor of VCAM1 | Angiogenesis  Endothelium  Calcium | [18] |
| ENSOARG00000012316 | PTTG1IP* | 0.76 | 1.59E-04 | 4.85E-02 | Activator of FGF | Angiogenesis  endothelium  Calcium | [19] |
| ENSOARG00000012715 | PTGIS | 0.73 | 1.25E-04 | 4.49E-02 | Prostaglandin I2  Potent vasodilator Inhibition of blood clot formation | Angiogenesis  Endothelium  Calcium | [20] |
| ENSOARG00000013623 | PPP3CA | -0.62 | 1.33E-04 | 4.49E-02 | Calcineurin  Calcium signaling  VEGF pathway | Angiogenesis  Endothelium  Calcium | [21] |
| ENSOARG00000006037 | BMPER | -0.91 | 7.99E-05 | 3.79E-02 | Inhibition of BMP function  Pro-angiogenic | Angiogenesis  Endothelium  Calcium | [22] |
| ENSOARG00000011962 | TRPM2 | -1.23 | 1.41E-05 | 1.50E-02 | Endothelial barrier function  Increase vascular permeability | Angiogenesis  Endothelium  Calcium | [23] |
| ENSOARG00000009518 | SOX4* | 0.64 | 7.50E-05 | 3.70E-02 | Associated with Wnt signaling | Differentiation  Development | [24] |
| ENSOARG00000011866 | PGAP6 | 0.62 | 1.33E-04 | 4.49E-02 | TMEM8A  Embryonic development | Differentiation  Development | [25] |
| ENSOARG00000009005 | C2CD3 | -0.54 | 8.82E-05 | 4.00E-02 | Associated with SHH signaling | Differentiation  Development | [26] |
| ENSOARG00000003978 | NOC3L | -0.55 | 7.41E-06 | 9.27E-03 | Adipogenesis | Differentiation  Development | [27] |
| ENSOARG00000019294 | ABCA12 | -1.13 | 3.81E-05 | 2.56E-02 | Lung development  ABCA12 KO leads to alveolar collapse | Differentiation  Development | [28] |
| ENSOARG00000020209 | CHPF | 0.86 | 5.82E-05 | 2.98E-02 | Chondroitin sulfate synthase-2  Regulation of ECM | ECM  Remodeling | [29] |
| ENSOARG00000008205 | IGSF8 | 0.66 | 1.16E-04 | 4.49E-02 | Negative regulator of TGF-beta | ECM  Remodeling | [30] |
| ENSOARG00000005315 | MMP1 | -1.06 | 1.43E-04 | 4.64E-02 | Breakdown of ECM | ECM  Remodeling | [31, 32] |
| ENSOARG00000005084 | MMP3 | -1.41 | 1.19E-06 | 2.17E-03 | Breakdown of ECM | ECM  Remodeling | [31, 32] |
| ENSOARG00000024340 | Metazoa_SRP | 1.85 | 2.47E-11 | 3.16E-07 | 7SL RNA  Non-coding RNA | Transcription regulation | [33] |
| ENSOARG00000023771 | miRNA* | 1.58 | 9.81E-09 | 4.19E-05 | Non-coding RNA | Transcription regulation |  |
| ENSOARG00000022840 | RNase_MRP | 1.58 | 6.06E-10 | 3.88E-06 | Ribonuclease | Transcription regulation | [34] |
| ENSOARG00000022831 | 5_8S_rRNA | 1.53 | 7.01E-08 | 2.24E-04 | 5.8S ribosomal RNA  Non-coding RNA | Transcription regulation |  |
| ENSOARG00000021831 | miRNA* | 1.43 | 3.88E-07 | 8.29E-04 | Non-coding RNA | Transcription regulation |  |
| ENSOARG00000023915 | RNaseP_nuc | 1.34 | 2.66E-06 | 4.26E-03 | Ribonuclease | Transcription regulation |  |
| ENSOARG00000023715 | 7SK RNA | 1.18 | 2.09E-05 | 1.81E-02 | Non-coding RNA | Transcription regulation | [35] |
| ENSOARG00000012889 | CPSF2 | -0.47 | 5.13E-05 | 2.74E-02 | mRNA processing  and polyadenylation | Transcription regulation | [36] |
| ENSOARG00000017276 | MAGE2* | 1.18 | 1.76E-05 | 1.73E-02 | MAGE-like protein  Protein trafficking and recycling | Secretory pathway & cytoskeleton | [37] |
| ENSOARG00000005241 | MARCHF9 | 0.92 | 4.86E-05 | 2.74E-02 | Protein processing | Secretory pathway & cytoskeleton | [38] |
| ENSOARG00000018257 | TRIM3 | 0.59 | 3.92E-05 | 2.56E-02 | BERP  Interact with myosins | Secretory pathway & cytoskeleton | [39, 40] |
| ENSOARG00000019076 | TBC1D20 | 0.41 | 1.67E-04 | 4.97E-02 | Inhibition of RAB1  Autophagosome maturation | Secretory pathway & cytoskeleton | [41] |
| ENSOARG00000011359 | MAP7D3* | -0.35 | 1.14E-04 | 4.49E-02 | Regulates microtubule assembly and stability | Secretory pathway & cytoskeleton | [42] |
| ENSOARG00000011149 | CRYBG1 | -0.89 | 1.27E-04 | 4.49E-02 | AIM1  Suppresor of cell migration | Secretory pathway & cytoskeleton | [43] |
| ENSOARG00000006047 | PLS1 | -1.18 | 5.12E-05 | 2.74E-02 | Plastin-1/Fimbrin  Cross-linking with actin  Formation of filopodia | Secretory pathway & cytoskeleton | [44] |
| ENSOARG00000012318 | HMG20B | 0.77 | 2.12E-05 | 1.81E-02 | DNA repair  Mitosis | Other | [45] |
| ENSOARG00000013664 | SKA3 | -1.13 | 1.14E-04 | 4.49E-02 | Mitosis | Other | [46] |
| ENSOARG00000019275 | BARD1 | -1.22 | 2.85E-05 | 2.14E-02 | DNA repair | Other | [47] |
| ENSOARG00000009025 | ZNF503* | 0.98 | 6.22E-06 | 8.85E-03 | Fanconi anemia | Other |  |
| ENSOARG00000009492 | COQ10A | 0.63 | 1.45E-04 | 4.64E-02 | Metabolism | Other |  |
| ENSOARG00000020345 | WDR53 | -0.35 | 1.21E-04 | 4.49E-02 | Unknown function | Other |  |
| ENSOARG00000000778 | MET | -0.78 | 9.06E-05 | 4.00E-02 | Receptor tyrosine kinase | Other |  |
| ENSOARG00000001759 | FANCL | -0.89 | 4.00E-05 | 2.56E-02 | Fanconi anemia | Other |  |
| ENSOARG00000004915 | TM4SF18 | -1.22 | 2.70E-05 | 2.14E-02 | Unknown function | Other |  |
| ENSOARG00000019309 | ATIC | -1.34 | 2.95E-07 | 7.56E-04 | Inosine monophosphate synthase | Other |  |

* Genes annotated manually.

1. Villenave R, Thavagnanam S, Sarlang S, Parker J, Douglas I, Skibinski G, et al. In vitro modeling of respiratory syncytial virus infection of pediatric bronchial epithelium, the primary target of infection in vivo. Proc Natl Acad Sci U S A. 2012;109(13):5040-5. Epub 2012/03/14. doi: 10.1073/pnas.1110203109. PubMed PMID: 22411804; PubMed Central PMCID: PMCPMC3323997.

2. Guohua Pana JHB, Valsala Haridasc, Shuxia Wangb, Ding Liub, Guoliang Yub, Claudius Vincenza, Bharat B. Aggarwalc, Jian Ni, Vishva M. Dixit. Identification and functional characterization of DR6, a novel death domain-containing TNF receptor. 1998.

3. Pan X, Jiang B, Liu J, Ding J, Li Y, Sun R, et al. STC1 promotes cell apoptosis via NF-κB phospho-P65 Ser536 in cervical cancer cells. Oncotarget. 2017;8(28):46249-61. Epub 2017/05/26. doi: 10.18632/oncotarget.17641. PubMed PMID: 28545028; PubMed Central PMCID: PMCPMC5542264.

4. Seo JY, Yaneva R, Cresswell P. Viperin: a multifunctional, interferon-inducible protein that regulates virus replication. Cell Host Microbe. 2011;10(6):534-9. Epub 2011/12/20. doi: 10.1016/j.chom.2011.11.004. PubMed PMID: 22177558; PubMed Central PMCID: PMCPMC3246677.

5. Gonzalez-Sanz R, Mata M, Bermejo-Martin J, Alvarez A, Cortijo J, Melero JA, et al. ISG15 Is Upregulated in Respiratory Syncytial Virus Infection and Reduces Virus Growth through Protein ISGylation. J Virol. 2016;90(7):3428-38. Epub 2016/01/15. doi: 10.1128/JVI.02695-15. PubMed PMID: 26763998; PubMed Central PMCID: PMCPMC4794669.

6. Berry KN, Kober DL, Su A, Brett TJ. Limiting Respiratory Viral Infection by Targeting Antiviral and Immunological Functions of BST-2/Tetherin: Knowledge and Gaps. Bioessays. 2018;40(10):e1800086. Epub 2018/08/17. doi: 10.1002/bies.201800086. PubMed PMID: 30113067; PubMed Central PMCID: PMCPMC6371793.

7. Busse DC, Habgood-Coote D, Clare S, Brandt C, Bassano I, Kaforou M, et al. Interferon-Induced Protein 44 and Interferon-Induced Protein 44-Like Restrict Replication of Respiratory Syncytial Virus. J Virol. 2020;94(18). Epub 2020/07/03. doi: 10.1128/JVI.00297-20. PubMed PMID: 32611756; PubMed Central PMCID: PMCPMC7459546.

8. Behera AK, Kumar M, Lockey RF, Mohapatra SS. 2'-5' Oligoadenylate synthetase plays a critical role in interferon-gamma inhibition of respiratory syncytial virus infection of human epithelial cells. J Biol Chem. 2002;277(28):25601-8. Epub 2002/05/01. doi: 10.1074/jbc.M200211200. PubMed PMID: 11980899.

9. Yu M, Tong JH, Mao M, Kan LX, Liu MM, Sun YW, et al. Cloning of a gene (RIG-G) associated with retinoic acid-induced differentiation of acute promyelocytic leukemia cells and representing a new member of a family of interferon-stimulated genes. Proceedings of the National Academy of Sciences of the United States of America. 1997;94(14):7406-11. Epub 1997/07/08. doi: 10.1073/pnas.94.14.7406. PubMed PMID: 9207104; PubMed Central PMCID: PMCPMC23834.

10. Ribaudo M, Barik S. The nonstructural proteins of Pneumoviruses are remarkably distinct in substrate diversity and specificity. Virol J. 2017;14(1):215. Epub 2017/11/08. doi: 10.1186/s12985-017-0881-7. PubMed PMID: 29110727; PubMed Central PMCID: PMCPMC5674761.

11. Oudshoorn D, van Boheemen S, Sánchez-Aparicio MT, Rajsbaum R, García-Sastre A, Versteeg GA. HERC6 is the main E3 ligase for global ISG15 conjugation in mouse cells. PloS one. 2012;7(1):e29870. Epub 2012/01/25. doi: 10.1371/journal.pone.0029870. PubMed PMID: 22272257; PubMed Central PMCID: PMCPMC3260183.

12. Kalinowski A, Galen BT, Ueki IF, Sun Y, Mulenos A, Osafo-Addo A, et al. Respiratory syncytial virus activates epidermal growth factor receptor to suppress interferon regulatory factor 1-dependent interferon-lambda and antiviral defense in airway epithelium. Mucosal Immunol. 2018;11(3):958-67. Epub 2018/02/08. doi: 10.1038/mi.2017.120. PubMed PMID: 29411775; PubMed Central PMCID: PMCPMC6431552.

13. DeDiego ML, Martinez-Sobrido L, Topham DJ, Williams BRG. Novel Functions of IFI44L as a Feedback Regulator of Host Antiviral Responses. Journal of Virology. 2019;93(21). doi: 10.1128/jvi.01159-19.

14. Parker N, Porter AC. Identification of a novel gene family that includes the interferon-inducible human genes 6-16 and ISG12. BMC Genomics. 2004;5(1):8. Epub 2004/01/20. doi: 10.1186/1471-2164-5-8. PubMed PMID: 14728724; PubMed Central PMCID: PMCPMC343271.

15. Antigny F, Girardin N, Frieden M. Transient receptor potential canonical channels are required for in vitro endothelial tube formation. J Biol Chem. 2012;287(8):5917-27. Epub 2011/12/29. doi: 10.1074/jbc.M111.295733. PubMed PMID: 22203682; PubMed Central PMCID: PMCPMC3285360.

16. Shibuya M. Vascular Endothelial Growth Factor (VEGF)-Receptor2: Its Biological Functions, Major Signaling Pathway, and Specific Ligand VEGF-E. Endothelium. 2009;13(2):63-9. doi: 10.1080/10623320600697955.

17. Han S-W, Jung Y-K, Lee E-J, Park H-R, Kim G-W, Jeong J-H, et al. DICAM inhibits angiogenesis via suppression of AKT and p38 MAP kinase signalling. Cardiovascular Research. 2013;98(1):73-82. doi: 10.1093/cvr/cvt019.

18. K Hibi KY, R Ueda, Y Horio, Y Murata, M Tamari, K Uchida, T Takahashi, Y Nakamura, T Takahashi. Aberrant upregulation of a novel integrin alpha subunit gene at 3p21.3 in small cell lung cancer. Oncogene. 1994.

19. Reynolds LE, Watson AR, Baker M, Jones TA, D'Amico G, Robinson SD, et al. Tumour angiogenesis is reduced in the Tc1 mouse model of Down's syndrome. Nature. 2010;465(7299):813-7. Epub 2010/06/11. doi: 10.1038/nature09106. PubMed PMID: 20535211; PubMed Central PMCID: PMCPMC3479956.

20. Wang XJ, Xu XQ, Sun K, Liu KQ, Li SQ, Jiang X, et al. Association of Rare PTGIS Variants With Susceptibility and Pulmonary Vascular Response in Patients With Idiopathic Pulmonary Arterial Hypertension. JAMA Cardiol. 2020;5(6):677-84. Epub 2020/04/03. doi: 10.1001/jamacardio.2020.0479. PubMed PMID: 32236489; PubMed Central PMCID: PMCPMC7113838.

21. Wang K, Song Y, Chen DB, Zheng J. Protein phosphatase 3 differentially modulates vascular endothelial growth factor- and fibroblast growth factor 2-stimulated cell proliferation and signaling in ovine fetoplacental artery endothelial cells. Biol Reprod. 2008;79(4):704-10. Epub 2008/05/30. doi: 10.1095/biolreprod.108.068957. PubMed PMID: 18509162; PubMed Central PMCID: PMCPMC2574765.

22. Heinke J, Wehofsits L, Zhou Q, Zoeller C, Baar KM, Helbing T, et al. BMPER is an endothelial cell regulator and controls bone morphogenetic protein-4-dependent angiogenesis. Circ Res. 2008;103(8):804-12. Epub 2008/09/13. doi: 10.1161/CIRCRESAHA.108.178434. PubMed PMID: 18787191.

23. Claudie M. Hecquet GUA, Asrar B. Malik. TRPM2 Channel Regulates Endothelial Barrier Function.

24. Pan B, Xue X, Zhang D, Li M, Fu J. SOX4 arrests lung development in rats with hyperoxia‑induced bronchopulmonary dysplasia by controlling EZH2 expression. International Journal of Molecular Medicine. 2017. doi: 10.3892/ijmm.2017.3171.

25. Lee GH, Fujita M, Takaoka K, Murakami Y, Fujihara Y, Kanzawa N, et al. A GPI processing phospholipase A2, PGAP6, modulates Nodal signaling in embryos by shedding CRIPTO. J Cell Biol. 2016;215(5):705-18. Epub 2016/11/25. doi: 10.1083/jcb.201605121. PubMed PMID: 27881714; PubMed Central PMCID: PMCPMC5147002.

26. Hoover AN, Wynkoop A, Zeng H, Jia J, Niswander LA, Liu A. C2cd3 is required for cilia formation and Hedgehog signaling in mouse. Development. 2008;135(24):4049-58. Epub 2008/11/14. doi: 10.1242/dev.029835. PubMed PMID: 19004860; PubMed Central PMCID: PMCPMC3120044.

27. Tominaga K. Fad24, a mammalian homolog of Noc3p, is a positive regulator in adipocyte differentiation. Journal of Cell Science. 2004;117(25):6217-26. doi: 10.1242/jcs.01546.

28. Yanagi T, Akiyama M, Nishihara H, Sakai K, Nishie W, Tanaka S, et al. Harlequin ichthyosis model mouse reveals alveolar collapse and severe fetal skin barrier defects. Human Molecular Genetics. 2008;17(19):3075-83. doi: 10.1093/hmg/ddn204.

29. Yada T, Gotoh M, Sato T, Shionyu M, Go M, Kaseyama H, et al. Chondroitin sulfate synthase-2. Molecular cloning and characterization of a novel human glycosyltransferase homologous to chondroitin sulfate glucuronyltransferase, which has dual enzymatic activities. Journal of Biological Chemistry. 2003;278(32):30235-47. doi: 10.1074/jbc.M303657200.

30. Wang HX, Sharma C, Knoblich K, Granter SR, Hemler ME. EWI-2 negatively regulates TGF-beta signaling leading to altered melanoma growth and metastasis. Cell Res. 2015;25(3):370-85. Epub 2015/02/07. doi: 10.1038/cr.2015.17. PubMed PMID: 25656846; PubMed Central PMCID: PMCPMC4349253.

31. Moores RC, Brilha S, Schutgens F, Elkington PT, Friedland JS. Epigenetic Regulation of Matrix Metalloproteinase-1 and -3 Expression in Mycobacterium tuberculosis Infection. Front Immunol. 2017;8:602. Epub 2017/06/10. doi: 10.3389/fimmu.2017.00602. PubMed PMID: 28596772; PubMed Central PMCID: PMCPMC5442172.

32. Bonnans C, Chou J, Werb Z. Remodelling the extracellular matrix in development and disease. Nat Rev Mol Cell Biol. 2014;15(12):786-801. Epub 2014/11/22. doi: 10.1038/nrm3904. PubMed PMID: 25415508; PubMed Central PMCID: PMCPMC4316204.

33. VINCENT BOIVIN GD-F, SONIA COUTURE, RYAN M. NOTTINGHAM,, PHILIA BOUCHARD-BOURELLE AML, MICHELLE S. SCOTT, and SHERIF ABOU-ELELA. Simultaneous sequencing of coding and noncoding RNA reveals a human transcriptome dominated by a small number of highly expressed noncoding genes. 2018. doi: 10.1261/rna.064493.

34. Perederina A, Li D, Lee H, Bator C, Berezin I, Hafenstein SL, et al. Cryo-EM structure of catalytic ribonucleoprotein complex RNase MRP. Nat Commun. 2020;11(1):3474. Epub 2020/07/12. doi: 10.1038/s41467-020-17308-z. PubMed PMID: 32651392; PubMed Central PMCID: PMCPMC7351766.

35. Boivin V, Deschamps-Francoeur G, Couture S, Nottingham RM, Bouchard-Bourelle P, Lambowitz AM, et al. Simultaneous sequencing of coding and noncoding RNA reveals a human transcriptome dominated by a small number of highly expressed noncoding genes. Rna. 2018;24(7):950-65. Epub 2018/04/29. doi: 10.1261/rna.064493.117. PubMed PMID: 29703781; PubMed Central PMCID: PMCPMC6004057.

36. Zhang Y, Sun Y, Shi Y, Walz T, Tong L. Structural Insights into the Human Pre-mRNA 3'-End Processing Machinery. Mol Cell. 2020;77(4):800-9.e6. Epub 2019/12/08. doi: 10.1016/j.molcel.2019.11.005. PubMed PMID: 31810758; PubMed Central PMCID: PMCPMC7036032.

37. Tacer KF, Potts PR. Cellular and disease functions of the Prader-Willi Syndrome gene MAGEL2. Biochem J. 2017;474(13):2177-90. Epub 2017/06/20. doi: 10.1042/BCJ20160616. PubMed PMID: 28626083; PubMed Central PMCID: PMCPMC5594744.

38. Hoer S, Smith L, Lehner PJ. MARCH-IX mediates ubiquitination and downregulation of ICAM-1. FEBS Lett. 2007;581(1):45-51. Epub 2006/12/19. doi: 10.1016/j.febslet.2006.11.075. PubMed PMID: 17174307.

39. Li WW, Nie Y, Yang Y, Ran Y, Luo WW, Xiong MG, et al. Ubiquitination of TLR3 by TRIM3 signals its ESCRT-mediated trafficking to the endolysosomes for innate antiviral response. Proceedings of the National Academy of Sciences of the United States of America. 2020;117(38):23707-16. Epub 2020/09/04. doi: 10.1073/pnas.2002472117. PubMed PMID: 32878999; PubMed Central PMCID: PMCPMC7519224.

40. El-Husseini AE, Vincent SR. Cloning and characterization of a novel RING finger protein that interacts with class V myosins. The Journal of biological chemistry. 1999;274(28):19771-7. Epub 1999/07/03. doi: 10.1074/jbc.274.28.19771. PubMed PMID: 10391919.

41. Sidjanin DJ, Park AK, Ronchetti A, Martins J, Jackson WT. TBC1D20 mediates autophagy as a key regulator of autophagosome maturation. Autophagy. 2016;12(10):1759-75. Epub 2016/08/04. doi: 10.1080/15548627.2016.1199300. PubMed PMID: 27487390; PubMed Central PMCID: PMCPMC5079675.

42. Yadav S, Verma PJ, Panda D. C-terminal region of MAP7 domain containing protein 3 (MAP7D3) promotes microtubule polymerization by binding at the C-terminal tail of tubulin. PloS one. 2014;9(6):e99539. Epub 2014/06/14. doi: 10.1371/journal.pone.0099539. PubMed PMID: 24927501; PubMed Central PMCID: PMCPMC4057234.

43. Haffner MC, Esopi DM, Chaux A, Gurel M, Ghosh S, Vaghasia AM, et al. AIM1 is an actin-binding protein that suppresses cell migration and micrometastatic dissemination. Nat Commun. 2017;8(1):142. Epub 2017/07/28. doi: 10.1038/s41467-017-00084-8. PubMed PMID: 28747635; PubMed Central PMCID: PMCPMC5529512.

44. de Arruda MV, Watson S, Lin CS, Leavitt J, Matsudaira P. Fimbrin is a homologue of the cytoplasmic phosphoprotein plastin and has domains homologous with calmodulin and actin gelation proteins. J Cell Biol. 1990;111(3):1069-79. Epub 1990/09/01. doi: 10.1083/jcb.111.3.1069. PubMed PMID: 2391360; PubMed Central PMCID: PMCPMC2116281.

45. Marmorstein LY, Kinev AV, Chan GK, Bochar DA, Beniya H, Epstein JA, et al. A human BRCA2 complex containing a structural DNA binding component influences cell cycle progression. Cell. 2001;104(2):247-57. Epub 2001/02/24. doi: 10.1016/s0092-8674(01)00209-4. PubMed PMID: 11207365.

46. Hou Y, Wang Z, Huang S, Sun C, Zhao J, Shi J, et al. SKA3 Promotes tumor growth by regulating CDK2/P53 phosphorylation in hepatocellular carcinoma. Cell Death Dis. 2019;10(12):929. Epub 2019/12/06. doi: 10.1038/s41419-019-2163-3. PubMed PMID: 31804459; PubMed Central PMCID: PMCPMC6895034.

47. Feki A, Jefford CE, Berardi P, Wu JY, Cartier L, Krause KH, et al. BARD1 induces apoptosis by catalysing phosphorylation of p53 by DNA-damage response kinase. Oncogene. 2005;24(23):3726-36. Epub 2005/03/23. doi: 10.1038/sj.onc.1208491. PubMed PMID: 15782130.
