# Supplementary material for: Pulmonary mesenchymal stem cells are engaged in distinct steps of host response to respiratory syncytial virus infection
Source: PLoS Pathog. 2021 Jul 28;17(7):e1009789. doi: 10.1371/journal.ppat.1009789 (PMC8351988; doi:10.1371/journal.ppat.1009789)
Supplement: S2 Table — (DOCX) [file ppat.1009789.s009.docx]

| **Patient ID** | **Age (months)** | **Pathology** |
| --- | --- | --- |
| PL002 | 143 | CPAM |
| PL003 | 10 | Congenital lobar over inflation |
| PL004 | 153 | Chronic bronchiolitis/pneumonia |
| PL005 | 5 days | Bronchial atresia |
| PL006 | 11 | CPAM |
| PL021 | 6 | CPAM |
| PL012 | 1.5 | Lobar emphysema |
| PL018 | 181 | Aspergilloma |
| PL009 | 5 | CPAM |

CPAM, congenital pulmonary airway malformation.
