# Supplementary material for: Pulmonary mesenchymal stem cells are engaged in distinct steps of host response to respiratory syncytial virus infection
Source: PLoS Pathog. 2021 Jul 28;17(7):e1009789. doi: 10.1371/journal.ppat.1009789 (PMC8351988; doi:10.1371/journal.ppat.1009789)
Supplement: S3 Table — (DOCX) [file ppat.1009789.s010.docx]

| **Gene** | **Primer** | **Sequence (5'-3')** | **Ref.** |
| --- | --- | --- | --- |
| 18S rRNA | FW  RV  P | CGCCGCTAGAGGTGAAATTC  GGCAAATGCTTTCGCTCTG  FAM-TGGACCGGCGCAAGACGGA-TAMRA | [1] |
| IFN-β | FW  RV  P | CGCCGCATTGACCATCTA  TTAGCCAGGAGGTTCTCAACAATAGTGTCA  FAM-TCAGACAAGATTCATCTAGCACTGGCTGGA-BHQ1 | [2] |
| IFN λ1 | FW  RV  P | GGACGCCTTGGAAGAGTCACT  AGAAGCCTCAGGTCCCAATTC  FAM-AGTTGCAGCTCTCCTGTCTTCCCCG-BHQ1 | [3] |
| RIG-I | FW  RV  P | CCAAGCCAAAGCAGTTTTCAA  CACATGGATTCCCCAGTCATG  FAM-TTGAAAAAAGAGCAAAGATATTCTGTGCCCGAC-TAMRA | [4] |
| MDA5 | FW  RV  P | GATTCAGGCACCATGGGAAGT  AGGCCTGAGCTGGAGTTCTG  FAM-GGGATGCTCTTGCTGCCACATTCTCTT-TAMRA | [4] |
| 2',5'-OAS | FW  RV  P | GATTCAGGCACCATGGGAAGT  AGGCCTGAGCTGGAGTTCTG  FAM-GGGATGCTCTTGCTGCCACATTCTCTT-TAMRA | [5] |
| MxA | FW  RV  P | CAGCACCTGATGGCCTATCAC  CATGAACTGGATGATCAAAGG  FAM-AGGCCAGCAAGCGCATCTCCAG-TAMRA | [5] |
| Viperin | FW  RV  P | CACAAAGAAGTGTCCTGCTTGGT  AAGCGCATATATTTCATCCAGAATAAG  FAM-CCTGAATCTAACCAGAAGATGAAAGACTCC-TAMRA | [5] |
| ICAM-1 | FW  RV  P | TGCAGACAGTGACCATCTACAGC  TCTGAGACCTCTGGCTTCGTC  FAM-TTCCGGCGCCCAACGTGATT-TAMRA | [6] |
| Nucleolin | FW  RV  P | TCGCGAAGGCAGGTAAAAA  CGACCTCTTCTCCACTGCTATCA  FAM-AAGGTGACCCCAAGAAAATGGCTCCTC-TAMRA | [7] |
| Annexin A2 | FW  RV  P | GTGAAGAGGAAAGGAACCGA  CTTGATGCTCTCCAGCATGT  - | [8] |
| TLR4 | FW  RV  P | CAGAGTTGCTTTCAATGGCATC  AGACTGTAATCAAGAACCTGGAGG  - | [9] |
| CX3CR1 | FW  RV  P | AGTGTCACCGACATTTACCTCC  AAGG CGGTAGTGAATTTGCAC  - | [10] |
| hRSV-A2 (L) | FW  RV  P | GAACTCAGTGTAGGTAGAATGTTTGCA  TTCAGCTATCATTTTCTCTGCCAAT  FAM-TTTGAACCTGTCTGAACATTCCCGGTT-TAMRA | [11] |
| RSV-A (N) | FW  RV  P | TGCTAAGACYCCCCACCGTAAC  GGATTTTTGCAGGATTGTTTATGA  C5CT6GC7CT87W7CA-BHQ1 | [12] |

FW, forward; RV, reverse; P, probe.

1. Alves MP, Guzylack-Piriou L, Juillard V, Audonnet JC, Doel T, Dawson H, et al. Innate immune defenses induced by CpG do not promote vaccine-induced protection against foot-and-mouth disease virus in pigs. Clin Vaccine Immunol. 2009;16(8):1151-7. Epub 2009/06/26. doi: 10.1128/CVI.00018-09. PubMed PMID: 19553550; PubMed Central PMCID: PMCPMC2725537.

2. Gielen V, Sykes A, Zhu J, Chan B, Macintyre J, Regamey N, et al. Increased nuclear suppressor of cytokine signaling 1 in asthmatic bronchial epithelium suppresses rhinovirus induction of innate interferons. J Allergy Clin Immunol. 2015;136(1):177-88 e11. Epub 2015/01/30. doi: 10.1016/j.jaci.2014.11.039. PubMed PMID: 25630941; PubMed Central PMCID: PMCPMC4541718.

3. Contoli M, Message SD, Laza-Stanca V, Edwards MR, Wark PA, Bartlett NW, et al. Role of deficient type III interferon-lambda production in asthma exacerbations. Nat Med. 2006;12(9):1023-6. Epub 2006/08/15. doi: 10.1038/nm1462. PubMed PMID: 16906156.

4. Slater L, Bartlett NW, Haas JJ, Zhu J, Message SD, Walton RP, et al. Co-ordinated role of TLR3, RIG-I and MDA5 in the innate response to rhinovirus in bronchial epithelium. PLoS Pathog. 2010;6(11):e1001178. Epub 2010/11/17. doi: 10.1371/journal.ppat.1001178. PubMed PMID: 21079690; PubMed Central PMCID: PMCPMC2973831.

5. Gielen V, Johnston SL, Edwards MR. Azithromycin induces anti-viral responses in bronchial epithelial cells. Eur Respir J. 2010;36(3):646-54. Epub 2010/02/13. doi: 10.1183/09031936.00095809. PubMed PMID: 20150207.

6. Takahiro Maeda MT, Hiroshi Kosugi, and Hidehiko Saito. Up-regulation ofcostimulatory/adhesion molecules by histone deacetylase inhibitors in acute myeloid leukemia cells. 2000.

7. Jerke U, Tkachuk S, Kiyan J, Stepanova V, Kusch A, Hinz M, et al. Stat1 nuclear translocation by nucleolin upon monocyte differentiation. PLoS One. 2009;4(12):e8302. Epub 2009/12/17. doi: 10.1371/journal.pone.0008302. PubMed PMID: 20011528; PubMed Central PMCID: PMCPMC2788426.

8. Jiang SL, Pan DY, Gu C, Qin HF, Zhao SH. Annexin A2 silencing enhances apoptosis of human umbilical vein endothelial cells in vitro. Asian Pac J Trop Med. 2015;8(11):952-7. Epub 2015/11/29. doi: 10.1016/j.apjtm.2015.10.006. PubMed PMID: 26614996.

9. Ebener S, Barnowski S, Wotzkow C, Marti TM, Lopez-Rodriguez E, Crestani B, et al. Toll-like receptor 4 activation attenuates profibrotic response in control lung fibroblasts but not in fibroblasts from patients with IPF. American journal of physiology Lung cellular and molecular physiology. 2017;312(1):L42-l55. Epub 2016/11/07. doi: 10.1152/ajplung.00119.2016. PubMed PMID: 27815256.

10. Anderson CS, Chu CY, Wang Q, Mereness JA, Ren Y, Donlon K, et al. CX3CR1 as a respiratory syncytial virus receptor in pediatric human lung. Pediatr Res. 2020;87(5):862-7. Epub 2019/11/15. doi: 10.1038/s41390-019-0677-0. PubMed PMID: 31726465; PubMed Central PMCID: PMCPMC7774023.

11. Lee DC, Harker JA, Tregoning JS, Atabani SF, Johansson C, Schwarze J, et al. CD25+ natural regulatory T cells are critical in limiting innate and adaptive immunity and resolving disease following respiratory syncytial virus infection. J Virol. 2010;84(17):8790-8. Epub 2010/06/25. doi: 10.1128/JVI.00796-10. PubMed PMID: 20573822; PubMed Central PMCID: PMCPMC2919030.

12. Essaidi-Laziosi M, Lyon M, Mamin A, Fernandes Rocha M, Kaiser L, Tapparel C. A new real-time RT-qPCR assay for the detection, subtyping and quantification of human respiratory syncytial viruses positive- and negative-sense RNAs. Journal of virological methods. 2016;235:9-14. Epub 2016/05/18. doi: 10.1016/j.jviromet.2016.05.004. PubMed PMID: 27180039.
