# Supplementary material for: Pulmonary mesenchymal stem cells are engaged in distinct steps of host response to respiratory syncytial virus infection
Source: PLoS Pathog. 2021 Jul 28;17(7):e1009789. doi: 10.1371/journal.ppat.1009789 (PMC8351988; doi:10.1371/journal.ppat.1009789)
Supplement: S4 Table — (DOCX) [file ppat.1009789.s011.docx]

| **Antibody** | **Dilution** (FCM, IF) | **Host, isotype** | **Clone** | **Reference, Source** |
| --- | --- | --- | --- | --- |
| Anti-CD73 | 1:200 | Mouse, IgG1 | AD2 | ab30451, abcam |
| Anti-CD90 | 1:100 | Mouse IgG1 | 5E10 | 555593, BD Pharmingen |
| Anti-CD105, FITC | 1:25 | Mouse, IgG2a | MEM-229 | ab53318, abcam |
| anti-RSV F protein | 1:100, 1:400 | Mouse, IgG2a | 131-2A | MAB8599, Millipore |
| Anti-RSV, biotin | 1:160 | Goat, IgG | - | 7950-0104, Bio-Rad |
| Cleaved Caspase-3 | 1:800 | Rabbit | Asp175 | 9661, Cell Signling |
| Anti-CD29 | 1:100 | Mouse, IgG1 | S-FW4-101 | S-BOV2034 WSU |
| Anti-CD44 | 1:100 | Mouse, IgG3 | S-BAG40A | S-BOV2037WSU |
| Anti-CD166, FITC | 1:20 | Mouse, IgG1 | 3A6 | MCA1926FT, Bio-Rad |
| Anti-CD31, FITC | 1:40 | Mouse, IgG2a | CO.3E1D4 | MA1-80360, Thermofisher |
| Anti-CD45, PE | 1:100 | Mouse, IgG1 | 1.11.32 | MCA2220PE, Bio-Rad |
| Anti-pan-cytokeratin, APC | 1:800 | Mouse, IgG1 | C-11 | MA1-10325, Thermofisher |
| Anti-Mouse IgG1, af488 | 1:2000 | Goat, IgG | - | A-21121, Thermofisher |
| Anti-Mouse IgG1, PE | 1:200 | Goat, IgG | - | A-21129, Thermofisher |
| Anti-Mouse IgG1, PerCP-Cy5.5 | 1:80 | Rat, IgG | RMG1-1 | 406612, Biolegend |
| Anti-Mouse IgG1, PE-Cy7 | 1:640 | Rat, IgG | RMG1-1 | 406614, Biolegend |
| Anti-Mouse IgG1, af647 | 1:1000 | Goat, IgG | - | A-21240, Thermofisher |
| Anti-Mouse IgG1, BV421 | 1:80 | Rat, IgG | RMG1-1 | 406616, Biolegend |
| Anti-Mouse IgG3, PerCP/Cy5.5 | 1:200 | Goat, IgG | - | 1100-13, Southernbiotech |
| Streptavidin, Pacific Blue | 1:200 | - | - | S11222, Thermofisher |
| Anti-Mouse IgG2a, af647 | 1:1000 | Goat, IgG | - | A-21241, Thermofisher |
| Anti-Mouse IgG2a, af546 | -, 1:400 | Goat, IgG | - | A-21133, ThermoFisher |
| Anti-Mouse IgG2a, af488 | -, 1:200 | Goat, IgG | - | A-21131, ThermoFisher |
| Wheat Germ Agglutinin, af633 | -, 1:200 | - | - | W21404, ThermoFisher |

FCM, flow cytometry; IF, immunofluorescence
