# Supplementary material for: Pulmonary mesenchymal stem cells are engaged in distinct steps of host response to respiratory syncytial virus infection
Source: PLoS Pathog. 2021 Jul 28;17(7):e1009789. doi: 10.1371/journal.ppat.1009789 (PMC8351988; doi:10.1371/journal.ppat.1009789)
Supplement: Attachment — Submitted filename: Brugger et al_Reviewers Comments_Revisions.pdf [file ppat.1009789.s012.pdf]

We thank the Editorial Board of *PLOS Pathogens* for giving us the opportunity to submit a revised version of our manuscript. With the help of the Reviewer's comments/suggestions and additional experiments, we think that the quality of our manuscript has significantly improved. Please find below our point-to-point answers to each comment in blue print. All the changes made in the original manuscript are also highlighted in blue print.

The reviewers raised very fundamental issues with the virology presented in this paper. While there is interest, you must provide stronger evidence of virus replication and virus spread. The concerns raised and experiments requested by Reviewer 1 and echoed by Reviewer 3 must be addressed.

We have addressed the concerns of the Reviewers by performing additional experiments to fully demonstrate the replication and spread of RSV in LR-MSCs (please see "Part I to III" for extensive replies).

PART I, SUMMARY

Reviewer 1

1. Brugger et al. have examined the response to respiratory syncytial virus (RSV) of lung-resident (LR) mesenchymal stem and stromal cells (MSCs) in both primary differentiated pediatric MSCs and in vivo, in the lamb model of RSV infection. MSCs are thought to play a role in the alveolar niche as regulators of homeostasis and regeneration. The transcriptional response to RSV of LR-MSCs initiates with an antiviral signature but later switches to repair mechanisms of differentiation, tissue remodeling, and angiogenesis.

The investigators state (l.45) that "[i]n the alveolar niche, LR-MSCs can interact with epithelial cells (AECs), which are the primary cellular target of most respiratory viruses [12-14]." In fact, these three references all refer to airway epithelial cells, the epithelial cells in the small airways, not the alveolar epithelial cells. RSV's target cell is the airway epithelial ciliated cells, as is influenza virus. If RSV does infect alveolar cells, that needs to be shown. This report does not, nor does it cite another report that does. Alveolar cells are very different from airway epithelial cells. And the premise (l.36) that "[l]ung-resident (LR)-MSCs can promote alveolar cell growth, differentiation, and self-renewal" would not seem to be relevant if the cells that are damaged by RSV are the airway epithelial cells, not the alveolar epithelial cells.

We agree with the Reviewer and are sorry for the mix-up. We made now a clear distinction throughout the manuscript between airway epithelial cells (AECs), as primary target of RSV

infection, and of alveolar cells (pneumocytes) which represent a secondary target for RSV infection and key in the pathogenesis of severe RSV disease. Also, we have added references where MSCs were identified in the nasal as well as in the bronchial tissue (references 4-6). Finally, we provide now experimental evidence of *in vivo* and *ex vivo* infection of alveolar cells by RSV (please see our replies to points 8. and 18. and also the new data presented in Figures 3C and S3D, E).

2. As described above, if the MSC cells are not a natural target cell for RSV, they may not express the RSV receptor, that might account for the poor efficiency of infection in vitro. The authors examined the MSCs for transcripts for every suggested receptor reported for RSV on immortalized cell lines, but not the one that is considered the most likely in vivo receptor, CX3CR1. CX3CR1 has been shown to be the receptor on ciliated epithelial cells in the airway epithelium.

Assuming that the frequency of RSV-positive LR-MSCs is rapidly increasing over time following infection of LR-MSCs (Figure 1E), we can reasonably state that LR-MSCs are "highly permissive towards RSV infection". Nevertheless, please see our following replies to points 3. to 7. for additional experiments supporting this statement. As requested by the Reviewer, we have measured the expression of CX3CR1 in matched AECs and LR-MSCs and similarly to the other putative RSV surface receptors tested, CX3CR1 is expressed in both cell types (Ct values of 28-30 cycles). The results of CX3CR1 levels are presented in the updated Figure 1C. The results and the method sections have been updated accordingly.

3. The investigators state (l.78) that they "detected fast replication kinetics of RSV in LR-MSCs similar to levels measured in infected AECs (Fig. 1D)." They state that "after 24 to 48 hours post-infection (p.i.) around 80% of LR-MSCs were infected by RSV-A2." But they show no evidence for that claim. Fig. 1D It shows the number of RSV genome copies, not the number of infected cells.

For some reason, the Reviewer is not referring to Figure 1E for whom the statement "after 24 to 48 hours post-infection (p.i.) around 80% of LR-MSCs were infected by RSV-A2" is referring to. Assuming the importance of these results to support replication of RSV in LR-MSCs, we decided to perform new infection studies in LR-MSCs from several donors by using the same MOIs for both RSV strains (0.1 and 1 PFU/cell). Indeed, in the previous version of the results we didn't compare both RSV strain in a consistent manner (different MOIs for each strain). The new results are presented in the updated Figures 1E and S1A and are described as follow:

Page 5, line 78-83: “To confirm the replication of LR-MSC, we next used a flow cytometry (FCM) approach. We infected LR-MSCs with a clinical isolate of RSV subtype A (RSV-ON1-H1) and with RSV-A2 at low multiplicity of infection (MOI; 0.1 PFU/cell). After 144 hours post-infection (p.i.) 40-100% of LR-MSCs were infected depending on the donor (**Fig. 1E**). However, when using higher MOIs (1 PFU/cell), 144 hours p.i., both RSV-A2 and RSV-ON1-H1 infected nearly 100% of the cells (**Fig. S1A**)”.

4. When they do show a picture of infected cells (Fig. 1F), only 2 cells in the field of probably 200 or more cells are infected at 36 hr and possibly 2 more by 48 hr. This infection was very inefficient, and the virus did not spread from cell to cell much at all. They do not mention what moi was used in this experiment in the text, but the Fig legend says moi of 0.1 to 0.5, which should result in the infection of 10-40% of the cells by 24 hr. That has not happened. Instead one of the infected cells has fused with many of its neighbors. The mechanism of syncytia formation does not require virus production, it only requires that the F protein reach the cell surface in its cleaved form where it can cause fusion of that cell’s membrane with that of its neighbor. In other words, this represents one infected cell. The other major cell also looks like a syncytium, though smaller.

We agree with the Reviewer that this scenario is among the possible explanations. To support our interpretation, we have performed additional infection experiments of LR-MSC and measured the intracellular infectious virus titers over time. These new data are presented in Figure 1I and S1C and indicate an exponential increase of intracellular infectious titers over time. Assuming the neglectable levels of infectious virus in the supernatants of infected LR-MSCs (now presented in Figure 1H and S1B), we think these data are supporting the concept of cell-to-cell spread of RSV in LR-MSCs. The text has been adapted accordingly (Page 6, line 98-104). Of note, the micrographs presented in Figure 1F were performed using a RSV-mCherry construct combined with a live imaging instrument allowing the monitoring of the mCherry reporter over time in a defined area in the cell layer (Nikon BioStation CT). Our experimental approach is clarified as follow: Page 5, line 84-7: “To follow visually virus spread in the two different cell types, we performed live imaging in specific areas of the cellular layer following infection with a recombinant RSV construct expressing constitutively the mCherry reporter (RSV-mCherry)”. In order to exclude the possibility that the RSV-mCherry-positive LR-MSCs appearing as foci (Figure 1F, lower panels) are due to “diffusion/dilution” of the mCherry within a syncytium, we decided to infect LR-MSCs from 2 independent donors with RSV-A2 (no mCherry reporter) combined with a high definition confocal microscopy approach. These new data presented in Figure 1G, show an increase of RSV signal from 24 to 48h p.i.

suggesting replication/spread of RSV. We have updated the text accordingly (Page 6, line 89-94).

5. The authors go on to show in Fig 1G and H that no infectious virus is released from the MSCs. That would explain the results in the Fig. 1F picture in which the virus did not spread from cell to cell, except by syncytia formation. Clearly, no virus is produced from the few MSCs that were initially infected. However, the syncytia could have produced many copies of the RSV genome as were detected in Figs 1C and 1D.

We kindly refer the Reviewer to our above reply to point 4.

6. Such poor infectivity and low-level virus production with fusion between the few infected cells and their neighbors is reminiscent of infection of differentiated airway cultures with RSV whose attachment protein, G, had been deleted. That virus infects ciliated cells between 1% and 0.1% as efficiently as RSV expressing G does. If these MSC cells do not express the receptor for RSV, the same type of low level of infection and low yield of virus would be expected probably, mediated by the RSV F protein instead of its G protein, accounting for the low level of infection, the syncytia formation and the lack of virus spread from cell to cell in the culture.

We kindly refer the Reviewer to our above reply to points 3. and 4.

7. The investigators conclude that “Altogether, these results demonstrate that primary pediatric LR-MSCs are highly permissive to RSV infection…” I would conclude the opposite.

We kindly refer the Reviewer to our above reply to points 3. and 4.

8. Fig 2 compares the gene expression of MSCs and HAEs when inoculated in vitro by RSV, at both transcripts produced (A and B) and proteins secreted (C – F), showing all kinds of responses. Again, the HAEs are not alveolar epithelial cells.

We agree with the Reviewer. However, primary human AECs culture at the air-liquid interface represents the gold standard for replication studies of RSV and other respiratory viruses. Thus, we selected this cell type as a control/reference when analyzing RSV replication in LR-MSCs. We have now clarified in the text the distinction between AECs and alveolar cells and we kindly refer the Reviewer to our reply to point 1.

9. There is the question, since RSV infects the small airway epithelial ciliated cells, how would the RSV reach the MSCs. Are there MSCs beneath the ciliated airway cells? The airway epithelium has tight junctions between cells and RSV is known to be exclusively shed apically (into the lumen of the airway). It is possible though that late in infection, if more of the airway epithelial cells are killed than can be replaced, the barrier may breakdown and allow virus shed into the lumen to leak through that barrier and to contact and infect underlying cells. If these underlying cells include MSCs and the infected MSCs produce mediators as shown in Fig. 2, those mediators might act on the alveolar epithelial cells that would be nearby. Is this what the authors are envisioning?

We provide now *in vivo* and *ex vivo* evidence of RSV infection of pneumocytes (please see our reply to point 18. and also, the new Figures 3C and S3D, E). We have discussed the potential mechanism of LR-MSC infection *in vivo* as follow: Page 14, line 314-8: “Since LR-MSCs are described to localize perivascular and in close proximity to the respiratory and alveolar epitheliums, this makes them a potential nonepithelial target for respiratory virus infection. Here, we show accessibility of LR-MSCs possibly through physical disruption of the alveolar epithelium. Indeed, already 3 days p.i., we observed evidence of lung injury associated with alveolar infection and concomitant LR-MSCs targeting by the virus.”.

10. The investigators then switch to their in vivo model of RSV infection in lambs inoculated with a very large dose of RSV, 108 pfu. They describe the shedding of RSV into the BAL and lung tissue which peaks around day 6, and the resolution of the infection over time in Fig. 3. They examined cells from the lung by FCM, which is not defined, but likely to be flow cytometry.

We agree with the Reviewer concerning the dose of RSV used for the experimental infection of lambs. The RSV dose per animal was selected in the range used by the Authors who established the lamb model of RSV infection (Group of Prof. Ackerman, Ohio State University, USA). Notably, in the literature, the inoculum of RSV used in animal experiments spans from 10^3 to 10^8 PFU depending on the species (ferrets, calves, lambs, rodents or non-human primates). This aspect is well covered in a recent review article by Taylor *et al.* (PMID 27908639). Also, we have now defined the abbreviation for “FCM” which is indeed “flow cytometry” appearing for the first time when describing the results of Figure 1E as follow: Page 5, line 78-83: “To confirm the replication of RSV in LR-MSC, we next used a flow cytometry (FCM) approach...”.

11. They found (l.158) "…at 6 days p.i. few syncytial cells lining alveoli were present." It is not clear if that means that "a" few syncytial cells were present or that none were present. That should be clarified. The lack of alveolar cell syncytia would be consistent with the lack of RSV infection. They present microscopic cross-sections the lung tissue and point to what they call syncytia, but it is not clear what they are seeing that leads them to that conclusion. More importantly, the sections are not stained for RSV antigens to determine if alveolar cells are infected by RSV and could therefore be responsible for the syncytia. They should be.

We are sorry for the unclear description of our pathological findings. We were meaning "a few". This observation was part of the pathological examination of the samples and was not designed to study RSV-induced syncytium formation *in vivo*. However, to prevent any misinterpretation, we have modified the text as follow: page 9, line 176-7: "In addition, at 6 days p.i. we observed occasionally potential syncytial cells lining alveoli (Fig. S4A, B).".

12. Ovine MSCs have been characterized before, but not lung resident MSCs (LR-MSCs) (l.136), which the investigators do here. They confirmed the multilineage capacity of the cells they isolated, a characteristic of MSCs. It is not clear why "ovine LR-MSCs transdifferentiated to chondrocytes, osteocytes, and adipocytes" would be relevant to repair of alveoli, but that is problem with the general concept of MSCs that is not unique to this report.

We fully agree with the Reviewer. To our knowledge there is no definitive evidence/proof that the *in vitro* trans-differentiation capacity of MSCs has any physiological relevance. We are using the capability of MSCs to differentiate towards chondro-, osteo-, and adipo-cytes as a criterion for MSC identity (together with plastic adherence and phenotypic markers expression) rather than an indication that this is occurring *in vivo*. Indeed, the trans-differentiation assay is part of the accepted indicators to identify MSCs. In order to avoid any misunderstanding, we have modified the text as follow: Page 8, line 156-8: "Together, these features fulfill the accepted criteria to identify MSCs proposed by the Mesenchymal and Tissue Stem Cell Committee of the International Society for Cellular Therapy [20].".

13. The investigators then examined pulmonary epithelial (CD31-CD45-panCTK+) and mesenchymal (CD31-CD45-panCTK-CD29+CD44+) compartments extracted from the lungs of lambs by bronchial epithelial lavage (BAL) and cultured. and detection of RSV in these cells. A micrograph in Fig. 4E has many yellow arrows pointing to a disturbance in the fibroblast monolayer which is not mentioned in the legend. It looks to be a syncytium. 4F shows a cluster

of nuclei that are very close to each other and could also be a syncytium but that is also not mentioned in the legend. The size of the nuclei in the top (from uninfected) and bottom (from infected lambs) pictures is quite different but the size bars are the same.

We thank the Review for the comment. We have now defined the arrows of Figure 4E in the Figure legend as follow: Page 43, line 1127: "The yellow arrowheads indicate a cluster of nuclei of a potential syncytium.". Surprisingly, the magnifications for the uninfected and infected cultures presented in Figure 4F are the same. We have clarified this aspect on the Figure legend as follow: Page 43, line 1030-1: "Scale bars, 15 µm (left and middle panels) and 10 µm for the 3D capture (right panels)". There is no information in the literature regarding nuclear swelling during syncytium formation but it is known that nuclear enlargement is a morphological feature of stress-induced premature senescence *in vitro* (PMIDs 18391457, 27340387) and a characteristic change of reactive cells to inflammatory signals (PMID 24563365). Interestingly, cells try to maintain constant the ratio of nuclear volume to cell volume, termed the "karyoplasmic ratio" (PMID 23277088). Assuming the size of the syncytium presented in Figure 4F compared to non-infected cells, this mechanism could maybe explain our findings. As highly speculative and out of the scope of the study we would not like to comment on this observation. However, if the Reviewer insists we will do so.

Reviewer #2

14. resident MSCs to the propagation and immune response to RSV infections. Using highly physiologically relevant models (pediatric human LR MSCs and lamb RSV infection model) authors, for the first time, demonstrated that MSCs are susceptible to RSV infections in vitro and in vivo. In vivo, analysis of transcriptional profile and CFU forming activity suggested that MSC play active role in modulation of anti-viral responses, lung repair and angiogenesis. Manuscript is well written, conclusions are supported by experimental evidence. I have no further questions and think that the manuscript can be published as is.

We are grateful that the Reviewer appreciates the quality of our study.

Reviewer #3

15. The authors reported a study on the response of lung-resident mesenchymal stem and stromal cells (LR-MSCs) to human RSV infection in a lamb model. They showed that primary pediatric LR-MSCs and LR-MSCs in the lamb model are permissive to RSV infection and also described the changes of their transcriptional profiles after RSV infection. The global

transcriptional response of LR-MSCs was shown to follow RSV disease, switching from an early antiviral signature to repair mechanisms including differentiation, tissue remodeling, and angiogenesis. This is an interesting finding, but to make the massage clear, the following comments should be considered.

We thank the Reviewer for the helpful recommendations that significantly improved the quality of our manuscript (please see Part II and Part III for further details).

PART II, MAJOR ISSUES: KEY EXPERIMENTS REQUIRED FOR ACCEPTANCE

Reviewer #1

16. The description of the RSV infection of in vitro-propagated MSCs must correlate with, rather than ignore the data. If syncytia are present, say so. If there is no virus produced and the infection does not spread to distant cells, don't claim that it does.

We hope that with the additional experiments performed and our replies to the Reviewer's concerns that we convincingly demonstrated that RSV is indeed replicating and spreading at high level in LR-MSCs. We kindly refer the Reviewer to our extensive replies to points 1. to 13.

17. In addition to testing for all the proposed in vitro cell RSV receptors by RT-PCR, they need to test for the only in vivo receptor that has been described.

We kindly reefer the Reviewer to our reply of point 2. presenting additional measurements of CX3CR1 levels in matched AECs and LR-MSCs.

18. Provide evidence that RSV infects alveolar epithelial cells in vivo, or that it does not. An H&E stained section of alveoli described as a syncytium without a clear evidence that it is a syncytium is not enough. The syncytia must be clear and RSV antigen must be found in the cell.

To demonstrate the infection of pneumocytes by RSV, we used two complementary and independent approaches. First, we performed immunohistochemistry stainings for RSV on lung sections of RSV-infected lambs. The results are presented in the new Figure 3C. We added portions of text accordingly: Page 9, lines 178-9: "Immunohistochemistry analysis of lung tissue sections revealed the presence of RSV antigen at 6 days p.i. in pneumocytes (Fig. 3C)". We updated the Methods section accordingly (Page 23, lines 589-97). Second, we performed *ex vivo* RSV-GFP infections on precision-cut lung slices (PCLS) generated from ovine lungs. These results appear in Figure S3D, E and are described in the results section as follow: Page 8, line 162-6: "Furthermore, *ex vivo* infection of ovine precision-cut lung slice (PCLS) cultures with a recombinant RSV construct expressing constitutively the green fluorescent protein (RSV-GFP) led to an increase of the reporter signal over time, indicating replication (**Fig. S3D**). When analyzing the infected PCLSs at higher magnification, the GFP signal was mainly located in the alveolar wall suggesting infection of pneumocytes (**Fig. S3E**)." A method section was added to describe the establishment of ovine PCLS cultures (Page 19,

Line 457-66). Additionally, the sections "RSV infection" and "Microscopy analysis" of the methods sections were adjusted.

Reviewer #2

(No Response)

Reviewer #3

19. Fig. 1G and H: It was shown that there was almost no infectious RSV detectable in supernatants of infected LR-MSCs, while LR-MSCs are susceptible to RSV infection as shown in Fig. 1D. I think this finding is very interesting and important. For better understanding, show the RSV copies number in the supernatants in addition.

In order to fully convince Reviewer 1 and 3 about the distinct mechanism of RSV replication in LR-MSCs in comparison to WD-AECs, we performed additional experiments. First, as requested by the Reviewer, we proceeded with the measurement of the extracellular viral RNA loads from infected WD-AECs and LR-MSCs. In line with the infectious virus release data (now presented in Figure 1H), we observed a rapid exponential increase of RSV loads in the supernatants of WD-AECs (increase of 10’000 times at 48 vs. 24h p.i.). However, in infected LR-MSC, we measured rather an accumulation of RSV RNA in the supernatants (increase of ca. 10 times at 48 vs 24h p.i.), suggesting the presence of substantial levels of defective (non-infectious) RSV particles. These data are presented in the new Figure 1J. Second, we decided to measure the intracellular infectious virus titers over time in LR-MSCs isolated from 3 independent donors. While the infectious RSV release was almost undetectable in infected LR-MSCs (Figure 1H, S1B), the intracellular infectious virus titers over time follow a typical virus growth curve, suggesting cell-to-cell spread of RSV infection in LR-MSCs (new data presented in Figure 1I). All these experiments were performed at two MOIs (0.1 and 1 PFU/cell). In order to prevent redundancy, we propose to move all the experiments using a MOI of 1 PFU/cell to the supplementary material (now present in Figure S1). We have adapted the main text in the results section accordingly (Page 6, line 98-105). Also, the Methods section and the Figure legends were updated.

20. Fig. 2E and F: From these results, the authors concluded that RSV infection leads to a robust activation of LR-MSCs, characterized by a strong antiviral and pro-inflammatory phenotype combined with cytokines modulating T cell function. Concerning this, the

involvement of the NS1 and NS2 proteins of RSV should be consider since these proteins are well known to be an IFN-antagonist inhibiting expression of antiviral host genes. It seems that the NSs function works well in WD-AECs but not in LR-MSCs.

We thank the Reviewer for this interpretation that we didn't' think about. To test this hypothesis, we measured over time the IFN-beta, IFN-lambda1 and -lambda2/3 mRNA levels in infected WD-AECs in comparison to LR-MSCs. The results, presented in the new Figure S2A-C, indicate similar IFN type I and III levels for both cell types, suggesting that the degree of NS1/2 inhibition mediated by RSV infection is comparable for both cell types. We have amended the results section (Page 7, line 123-5): "Notably, infection of WD-AECs in comparison to LR-MSCs induced comparable IFN type I and III levels 24 to 72 hours p.i., suggesting a similar IFN response upon RSV infection (Fig. S2A-C)". We would like to point that while going through the verification of all the results again, we identified a calculation mistake in the data of the IFN-λ1/3 immunoassay (Figure 2D). We made the correction and the new results are close to the previous ones and don't affect the interpretation of the results.

21. Fig. 6E: What is the RSV positive rate in LR-MSCs at 6 days? Does the transcriptional profile represent for RSV-infected LR-MSCs? There is a dramatic change with 14 days from 6 days. Was the expression of the genes changed in the same population of LR-MSCs? Or were these different populations? It is possible that the majority of RSV-infected LR-MSCs died before 14 days and uninfected LR-MSCs were newly generated. Discuss about the fate of RSV-infected LR-MSCs.

Our RNA-seq analysis of *ex vivo* expanded LR-MSCs revealed comparable transcriptional profiles for common markers used to identify MSCs, indicating isolation of the same cellular population at 6-, 14- and 42-days p.i (Fig.6A). Nonetheless, qPCR analysis showed that 6/8 LR-MSCs cultures, isolated from animals 6 days p.i., were positive for viral RNA (Fig. 4D). Therefore, it is likely that the transcriptional profile of these cells is representative for RSV-infected LR-MSCs. In line with this, both our *in vitro* (Fig. 2A-D) and *in vivo* (Fig. 6B) data indicate that RSV infection is shifting LR-MSCs towards an antiviral state.

As pointed by the Reviewer, LR-MSCs isolated from animals 14 days p.i. shows distinct profiles in comparison to 6 days p.i. This could represent a transitional state between virus clearance and repair/regeneration mechanisms. Indeed, there was no detectable viral RNA present in *ex vivo* expanded LR-MSCs at 14 days p.i (Fig. 4D) which is in line with the rare presence of viral RNA in the lung tissue 14 days p.i. (new Fig. 3D). We could observe some cytopathic effect of RSV during *in vitro* infection of LR-MSCs at late time points. (>4-5 days p.i.). While it is not possible to translate these observations to the *in vivo* situation, the

presence of RSV-positive multinucleated LR-MSCs in the BALs 3 days p.i. are indicative of a cytopathic effect of RSV on LR-MSCs (Fig. 4F). Also, following *in vivo* infection, the expansion of the pulmonary MSC compartment is suggesting a replenishment of the LR-MSC pool of RSV-injured lungs (Fig. 5C-F). We propose to discuss these aspects in the Discussion section as follow: Page 14, line 323-6: “During the acute phase of RSV disease, we observed RSV-positive multinucleated LR-MSCs in the BAL-expanded cultures, indicative of a cytopathic effect on these cells. Thereby, the concomitant expansion of the pulmonary MSC compartment is potentially indicative of a replenishment of the lost fraction of LR-MSCs following RSV infection.”.

PART III, MINOR ISSUES: EDITORIAL AND DATA PRESENTATION MODIFICATIONS

Reviewer #1

22. A more careful description and differentiation between airway epithelial cells and alveolar epithelial cells is needed. They are not the same, as is implied here (referencing literature on airway epithelial cells and claiming it support their idea of alveolar cell infection by RSV.

We thank the Reviewer for this important comment. We are now providing a clear distinction between airway and alveolar epithelial cells throughout the manuscript. We kindly reefer the Reviewer to our replies to points 1., 8., 9., and 18.

Reviewer #2

(No Response)

Reviewer #3

23. References: Lines 348-351 (MSCs are applied in cell-based therapies with promising outcomes for the treatment of pulmonary morbidities such as idiopathic pulmonary fibrosis, acute-respiratory distress syndrome and severe influenza infections [10, 351 56-58]): Suggest citing here below article: Yudhawati R, Amin M, Rantam FA, Prasetya RR, Dewantari JR, Nastri AM, Poetranto ED, Wulandari L, Lusida MI, Koesnowidagdo S, Soegiarto G, Shimizu YK, Mori Y, Shimizu K. Bone marrow-derived mesenchymal stem cells attenuate pulmonary inflammation and lung damage caused by highly pathogenic avian influenza A/H5N1 virus in BALB/c mice. BMC Infect Dis. 2020 Nov 11;20(1):823. doi: 10.1186/s12879-020-05525-2. PMID: 33176722; PMCID: PMC7656227. They reported that the administration of MSCs prevented further lung injuries and inflammation caused by a highly pathogenic avian influenza A/H5N1 virus, and enhanced alveolar cell type II and I regeneration.

As recommended by the Reviewer, we have added the cited reference to our manuscript. The article is appearing as reference 65.
